# Azithromycin Prevents Pregnancy Loss: Reducing the Level of Tumor Necrosis Factor-Alpha and Raising the Level of Interleukin-10 in Rats

**DOI:** 10.1155/2013/928137

**Published:** 2013-11-24

**Authors:** Ayse Er

**Affiliations:** Department of Pharmacology and Toxicology, Faculty of Veterinary Medicine, University of Selcuk, 42075 Konya, Turkey

## Abstract

The aim of this study was to determine the effect of azithromycin on LPS-induced pregnancy loss. Thirty-six pregnant female Wistar rats were divided into 4 equal groups as follows: control group, where 0.3 mL of normal saline solution was administered intravenously on day 10 of pregnancy; azithromycin group, where azithromycin was administered orally at 350 mg kg^−1^ day on days 9, 10, and 11 of pregnancy; lipopolysaccharide group, where LPS was administered intravenously via the tail vein at 160 **μ**g kg^−1^ on day 10 of pregnancy; and the azithromycin + LPS group, where azithromycin was administered orally at 350 mg kg^−1^ day on days 9, 10, and 11 of pregnancy and LPS was administered intravenously at 160 **μ**g kg^−1^ on day 10 of pregnancy. Blood samples were obtained from the tail vein on day 10 of the experiment. Pregnancy rates were determined. Tumor necrosis factor-alpha (TNF-**α**) and interleukin (IL-10) levels were measured by ELISA. Azithromycin prevented (*P* < 0.05) LPS-induced pregnancy loss. Higher TNF-**α** and IL-10 levels were measured (*P* < 0.05) in the LPS and azithromycin + LPS groups, respectively. In conclusion, azithromycin may be useful in infection- or endotoxemia-dependent pregnancy loss.

## 1. Introduction

The maternal immune system is capable of recognizing and refusing a response against foetal antigens [1]. One of the most common unfavorable outcomes during the first trimester of pregnancy is spontaneous abortion; the rate of spontaneous abortion is 15%–20% in women [2]. However, the mechanisms underlying pregnancy loss caused by maternal infections are not clear [3]. 

Lipopolysaccharide (LPS), an endotoxin derived from gram negative bacteria, has been used to constitute inflammatory response in experimental studies with pregnancy. It is well known as a trigger of abortion and preterm birth via proinflammatory cytokines and nitric oxide [4–9]. The predominant production of T-helper (Th2) cytokines is a characteristic of normal pregnancy, while a predominant production of Th1 cytokines is a characteristic of abortion and recurrent abortion [10]. A change from a Th2-biased to a Th1-biased cytokine profile in maternal serum results in complications for pregnant women, such as spontaneous abortions and preeclampsia [11].

Besides the direct antimicrobial effect, macrolides also show anti-inflammatory properties [12]. Azithromycin has similar efficacy compared with erythromycin or amoxicillin; azithromycin also has fewer adverse effects in the treatment of pregnant women with *Chlamydia trachomatis* infections [13]. Because of this feature, using azithromycin to treat pregnant women with uncomplicated *C. trachomatis *infections is increasing amongst obstetricians [14]. Azithromycin is clinically effective in the treatment of common respiratory, skin/skin-structure infections, nongonococcal urethritis, and cervicitis due to *C. trachomatis*. Azithromycin is categorized as a class B drug during pregnancy [15].

The balance between tumor necrosis factor-alpha (TNF-*α*) and interleukin (IL-10) is determined for pregnancy success. Whenever the level of TNF-*α* increases, abortion occurs, while IL-10 supports the pregnancy [10, 11]. Due to the inexistence of an adequate preventive treatment of early pregnancy loss, it has been hypothesized that azithromycin may prevent LPS-induced pregnancy loss because of an inhibitory effect on TNF-*α* and potentiating effect on IL-10.

The aim of this study was to determine the effect of azithromycin on LPS-induced pregnancy loss in rats. 

## 2. Materials and Methods

### 2.1. Animal

The study protocol was approved by the Ethical Committee of Necmettin Erbakan University, Experimental Medicine, Research and Application Center, Konya, Turkey. Thirty-six female and 9 male Wistar rats (272 ± 44.9 g and 306 ± 16.1 g, respectively, 5-6 months old) were used in this study. Rats were fed a standard pelleted diet and tap water was provided *ad libitum* as drinking water. Animals were bred in standard cages on a 12 hr light/dark cycle at room temperature in a humidity-controlled environment. 

### 2.2. Experimental Procedure

LPS (*Escherichia coli*, serotype O111:B4, Sigma-Aldrich Chemie, Deisenhofen, Germany) and azithromycin (Zitromax, 200 mg/5 mL, oral suspension, Pfizer, Istanbul, Turkey) were diluted with pyrogen-free saline to appropriate concentrations. 

Female rats were caged with males for 1 day and the presence of a vaginal plug was designated as day 0 of pregnancy. Tenth to 12th day in a rat's pregnancy corresponds roughly to the first trimester of human pregnancy [16]. Pregnant rats were randomly divided into 4 groups as follows: control group, where 0.3 mL of normal saline solution was administered intravenously on day 10 of pregnancy (*n* = 9); azithromycin group, where azithromycin was administered orally at 350 mg kg^−1^ day on days 9, 10, and 11 of pregnancy (*n* = 9); where LPS group, LPS was administered intravenously via the tail vein at 160 *μ*g kg^−1^ on day 10 of pregnancy (*n* = 9); and the azithromycin + LPS group, where azithromycin was administered orally at 350 mg kg^−1^ day on days 9, 10, and 11 of pregnancy and LPS was administered intravenously at 160 *μ*g kg^−1^ on day 10 of pregnancy (*n* = 9). Blood samples were obtained from the tail vein on day 10 of the experiment (3 hr after LPS administration) and all animals were followed during pregnancy. In addition, animals that did and did not give birth were determined. At the end of the study, all animals were euthanized under thiopental sodium anaesthesia (70 mg/kg, intraperitoneally; Pental Sodium 1 g inj., I. E. Ulagay Ilac Sanayi, Istanbul, Turkey).

### 2.3. Measurements

Samples were centrifuged and serum samples were stored at −70°C until analysis. TNF-*α* (eBioscience Rat TNF-*α* kit, sensitivity 11 pg/mL, San Diego, CA, USA) and IL-10 (eBioscience Rat IL-10kit, sensitivity 1.5 pg/mL, San Diego, CA, USA) levels were determined at 450 nm by commercial ELISA kits with ELISA reader (MWGt Lambda Scan 200, USA). 

### 2.4. Statistical Analysis

The pregnancy rates of the groups were evaluated using a chi-square test, and the concentrations of TNF-*α* and IL-10 were compared with ANOVA and the Tukey test. Data are expressed as the mean ± SE. Number of offspring in each group was evaluated by ANOVA and Duncan test. Significance was accepted at the *P* < 0.05 level. 

## 3. Results

**Figure 1:**
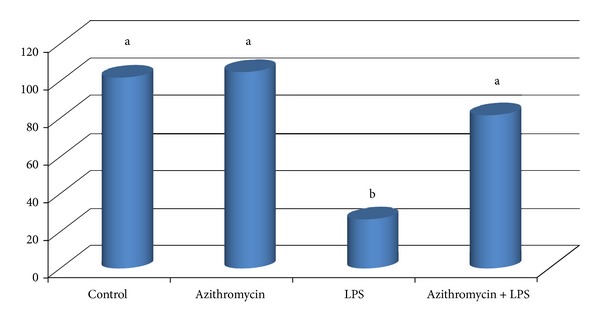
The number of offspring in groups. To induce the pregnancy loss with LPS, 160 *μ*g kg^−1^ LPS (*Escherichia coli* 0111:B4) was administered intravenously via the tail vein on day 10 of pregnancy in LPS group. Azithromycin was administered orally at 350 mg kg^−1^ day on days 9, 10, and 11 of pregnancy in azithromycin and azithromycin + LPS groups. Blood samples were obtained from the tail vein on day 10 of the experiment and all animals were followed during pregnancy. Animals that did and did not give birth were determined. ^a,b^Different letters are statistically significant (*P* < 0.05).

All animals were followed during pregnancy. The average weight of the rats was 272 ± 44.9 g before pregnancy and 375 ± 47.9 g at the end of pregnancy. Azithromycin inhibited (*P* < 0.05) LPS-induced pregnancy loss, and there were no adverse effects on the pregnancy rate (Table 1). The TNF-*α* level was higher (*P* < 0.05) in the LPS group, and the IL-10 levels were lower (*P* < 0.05) in the azithromycin and control groups (Table 2). In addition, the concentration of IL-10 in the azithromycin + LPS group was significantly higher (*P* < 0.05) than the other groups (Table 2). Offspring rate was statistically significant (*P* < 0.05) in LPS group when compared to all other groups (Figure 1). 

## 4. Comment

The aim of this study was to determine the effect of azithromycin on septic abortion. Many endogenous agents, such as prostaglandins or cytokines, play a pivotal role during pregnancy [10, 17]. Recurrent spontaneous abortion is classically defined as three or more pregnancy losses and usually occurs before 20 weeks of gestation. Recently, recurrent spontaneous abortion has been redefined as the spontaneous loss of two or more clinical pregnancies [18, 19]. Adverse pregnancy outcomes, such as spontaneous abortion, preterm labour, preeclampsia, and intrauterine growth restriction, can result from a deregulation of cytokines networks [20].

In the current study, azithromycin alone did not present a negative effect on the pregnancy rate (Table 1). It has been reported that the prophylactic use of azithromycin can decrease procedure-related pregnancy loss and may be safe in pregnant women [21, 22].

In the current study, higher pregnancy rates were determined in the control and azithromycin groups than in the LPS group (Table 1). In addition, a higher TNF-*α* level was measured in the LPS group (Table 2). The maternal immune response is determined for pregnancy success. Animal models have been used to elucidate this question. The mechanism underlying early pregnancy loss is associated with several inflammatory molecules; thus, modulation of the inflammatory modules is useful. Excessive inflammation may lead to unfavourable outcomes, such as spontaneous abortion and fetal resorption. Areas of implantation are extremely sensitive to LPS and Th1 cytokines (TNF-*α* and lL-2) during early pregnancy in mice. These molecules have the ability to induce embryonic resorption [3, 23, 24]. Low doses of LPS, without affecting mother survival, cause high rates of embryonic resorption during early pregnancy [3, 25]. Deregulation of cytokines networks results in adverse pregnancy outcomes [20]. Th2 cytokines, including IL-10, have a protective role, while Th1 cytokines, including TNF-*α*, are abortive factors in pregnancy [26]. Increased TNF-*α* levels caused by LPS resulted in insufficient placental perfusion, improvement of thrombotic events, and placental and fetal hypoxia [27]. In the current study, the LPS treatment increased TNF-*α* level, which determined a decrease in pregnancy rate. It has been reported that the LPS-increased TNF-*α* level is closely linked to recurrent pregnancy loss [28]. In another study, the concentration of LPS-binding protein in amniotic fluid was increased in patients who had a spontaneous fetal loss [29].

In the current study, azithromycin increased the pregnancy rate 3.5-fold when compared to the LPS group (Table 1). In addition, a higher IL-10 concentration occurred (*P* < 0.05) in the azithromycin + LPS group than those of the other groups, and the TNF-*α* level was lower (*P* < 0.05) in the azithromycin + LPS group than the LPS group (Table 2). Erythromycin and azithromycin are used in the treatment of endocervical chlamydial infections and mycoplasma pneumonia in obstetric patients [30]. Azithromycin may be more effective against endometrial infections because it provides important tissue levels for a long period [31]. Transplacental passage of azithromycin is limited, and azithromycin and other macrolide antibiotics are generally accepted to be safe in pregnancy [32, 33]. Increased pregnancy rates in the azithromycin + LPS group (Table 1) may reflect the depressive effect of macrolides on proinflammatory cytokines production and the potentiating effect on the IL-10 level. The suppressive effects of macrolides, including azithromycin, on the TNF-*α* level and the potentiating effects of these drugs on the IL-10 level have been reported [31, 34, 35]. Moreover, IL-10 injections prevent LPS-induced abortions and decrease LPS-induced fetal death [6, 27]. 

In conclusion, infection- or endotoxemia-mediated pregnancy loss may be prevented by using azithromycin during the pregnancy period.

## Figures and Tables

**Table 1:** Pregnancy rates of groups.

Drug	Pregnant/pregnancy loss	Labour animals
Control	9/0^a ^	9
Azithromycin	9/0^a^	9
LPS	9/7^b^	2
Azithromycin + LPS	9/2^a^	7

LPS: lipopolysaccharide (160 *µ*g kg^−1^ intravenously, *Escherichia coli* 0111:B4).^ a,b^Different letters in the same column are statistically significant (*P* < 0.05).

**Table 2:** TNF*α* and IL-10 levels of groups.

	TNF*α* (pg/mL)	IL-10 (pg/mL)
Control	ND	90.2 ± 4.31^a^
Azithromycin	58.2 ± 9.56^a^	113 ± 15.4^a^
LPS	128 ± 10.7^b^	496 ± 149^b^
Azithromycin + LPS	96.6 ± 10.2^c^	1130 ± 87.4^c^

TNF*α*: tumor necrosis factor *α*; IL-10: interleukin-10; LPS: lipopolysaccharide (160 *µ*g kg^−1^ intravenously, *Escherichia coli* 0111:B4). ND: not determined. ^a,b,c^Different letters in the same column are statistically significant (*P* < 0.05).
